# Body Composition and Serum Anti-Müllerian Hormone Levels in Euthyroid Caucasian Women With Hashimoto Thyroiditis

**DOI:** 10.3389/fendo.2021.657752

**Published:** 2021-07-29

**Authors:** Agnieszka Adamska, Anna Popławska-Kita, Katarzyna Siewko, Agnieszka Łebkowska, Anna Krentowska, Angelika Buczyńska, Łukasz Popławski, Piotr Szumowski, Małgorzata Szelachowska, Adam Jacek Krętowski, Irina Kowalska

**Affiliations:** ^1^Department of Endocrinology, Diabetology and Internal Medicine, Medical University of Białystok, Bialystok, Poland; ^2^Department of Internal Medicine and Metabolic Diseases, Medical University of Białystok, Bialystok, Poland; ^3^Department of Radiology, Medical University of Białystok, Bialystok, Poland; ^4^Department of Nuclear Medicine, Medical University of Białystok, Bialystok, Poland; ^5^Clinical Research Centre, Medical University of Białystok, Bialystok, Poland

**Keywords:** AMH, fat mass, leptin, Hashimoto autoimmune thyroiditis, BMI

## Abstract

**Objective:**

Women with Hashimoto thyroiditis (HT) are characterized by increased incidence of infertility and disturbances in body composition. Serum anti-Müllerian hormone (AMH), which reflects functional ovarian reserve, is decreased in women with HT and it be related to body mass. The aim of the present study was to investigate the relation between serum levels of AMH and body composition in HT compared to control group.

**Patients and Methods:**

We examined 85 euthyroid women: 39 subjects with HT and 46 control women. Body composition was analysed by dual-energy X-ray absorptiometry and with bioimpedance method. Serum concentrations of AMH, leptin, TSH, thyroid hormones were assessed.

**Results:**

We observed lower serum concentration of AMH in women with HT in comparison to the control group (p=0.01), but without differences in serum concentration of leptin between studied groups (p=0.28). Women with HT were characterized by higher %body fat (p=0.01) estimated with bioimpedance method without differences in BMI, android and gynoid fat mass and visceral adipose tissue (VAT) mass estimated with DXA method when compared to the control group (all p>0.05). We found a negative relationship between serum concentration of AMH and %body fat (r=-0.38,p=0.03) in women with HT. Additionally, in HT group, the relationship between serum levels of AMH and leptin was not statistically significant (r=0.01,p=0.96). We observed a relationship between serum concentration of leptin and BMI, %body fat mass, android, gynoid and VAT mass in HT and in the control group (all p<0.01).

**Conclusions:**

Women with HT are characterized by lower levels of AMH and it is associated with higher fat mass, independently of serum levels of leptin.

## Introduction

Ovarian reserve is defined as the number of oocytes remaining in the ovary, or oocyte quantity (oocyte number) which are the population of nongrowing, i.e., primordial, follicles. In turn, the quality of oocytes refers to fertilized oocyte potential to result in a live born infant (1). It has been proposed that the ovarian reserve should be the term used for defining the resting pool of follicles (i.e., the primordial follicles), and that the growing pool would be better defined as the functional ovarian reserve (2). The prevalence of diminished ovarian reserve in women is about 24% (3). It has been identified that smoking, previous ovarian surgery, pelvic irradiation, previous chemotherapy, as well as genetic causes are connected with diminished ovarian reserve (4). However, for most cases (approximately 90%), the specific reason of decreased ovarian reserve has not been found (5). The assessment of functional ovarian reserve includes the levels of follicle-stimulating hormone (FSH), anti-Müllerian hormone (AMH), inhibin B, and estradiol, and sonographically measured features of the ovaries, e.g. antral follicle count (AFC). These markers can be useful as predictors of oocyte yield following controlled ovarian stimulation and oocyte retrieval (1, 6).

Anti-Müllerian hormone is a glycoprotein involved in the regulation of follicle growth, inhibition of the recruitment of primordial follicle, and inhibition of aromatase expression, which leads to a decrease in granulosa cells sensitivity to FSH (7). This hormone is synthesized by the granulosa cells of growing preantral and early antral follicles but is not produced by primordial follicles (2). After an initial increase until early adulthood, AMH concentrations slowly decrease with increasing age until becoming undetectable when the stock of primordial follicles is exhausted. Individual AMH serum concentration accurately reflects the size of the pool of antral follicles and it has been shown that high serum level of AMH is associated with high AFC (8). Many studies have convincingly demonstrated that AMH is the best currently available measure of functional ovarian reserve (6, 9) and the best currently available indirect test in terms of sensitivity and specificity as opposed to AFC, FSH, E2 and inhibin B concentrations or various ovarian challenge tests (10). Additionally, it has been shown that serum concentration of AMH is connected with BMI in different clinical condition, although the data are inconsistent (11–13) and not focused on fat or fat free mass in HT group. In a prospective, cross-sectional study, involving 87 women with polycystic ovary syndrome (PCOS) and 67 non-PCOS women, inverse correlations between AMH and BMI, fat mass and waist circumference have been observed (14). However, in another study, serum levels of AMH were not associated with BMI, although they were connected with insulin resistance in PCOS women (15). Moreover it has been shown that obese women have lower AMH levels compared to non-obese women in the late reproductive years (16).

The diagnosis of Hashimoto thyroiditis (HT) is based on the presence of hypoechogenic structure of the thyroid gland in the USG and elevated serum concentration of thyroid peroxidase antibodies (TPOAbs) and/or antibodies against thyroglobulin (TgAbs) (17). To date, studies evaluating functional ovarian reserve in euthyroid women with HT or women with elevated TPOAbs are inconsistent (5). Some authors did not observe any association between serum levels of AMH and TPOAbs (5), whereas others found either higher levels of AMH in HT compared to the control group (12) or lower functional ovarian reserve in HT women *vs* controls (18). Nevertheless, the mechanisms related to the decreased functional ovarian reserve in patients with HT are not explained, although, could be connected with BMI (12, 13). Weight gain could therefore lead to a decrease in serum levels of AMH. This hypothesis could be supported by a study which has shown that hypocaloric diet, physical exercise and orlistat administration leads to weight loss and increase in AMH serum levels in overweight and obese women with polycystic ovary syndrome (19). To date, there is no available data focused on functional ovarian reserve and body composition (% of body fat as well as android fat mass, gynoid fat mass and visceral adipose tissue (VAT) mass) in euthyroid women with HT. A previous study has shown an association between subclinical and overt hypothyroidism and higher BMI (20), whereas the data focused on patients with HT in euthyreosis are obscure and indicate a relationship between serum concentration of fT3 and VAT (21). Adipose tissue is considered the primary site able to store energy excess but also an organ of endocrine secretion and secretes biologically active substances, including leptin (22, 23). It has been shown that leptin may play a role in the interaction between thyroid hormones and body composition (24) and leptin could be connected with thyroid autoimmunity (25) and has stimulatory effect on releasing TSH *in vivo* and has an impact on peripheral iodothyronine deiodinase activity and conversion of T4 to T3 (26). In the face of the fact that a positive association between serum levels of leptin or fat mass with the ratio of FT3 to FT4 was observed (24), it is important to examine the relationship of body composition with functional ovarian reserve and serum levels of leptin.

Therefore, taking into account the above-mentioned data, we investigated the relation between serum levels of AMH and leptin and body composition in euthyroid Caucasian women with HT in comparison to control group.

## Materials and Methods

### Ethic Approval

All the procedures used in the present study were performed in accordance with the 1964 Helsinki declaration and its later amendments and other relevant guidelines and regulations. The study was approved by the Institutional Review Board (Ethics Committee of Medical University of Białystok, Białystok, Poland, approval no. R-I-002/300/2015). The participation in the study was voluntary and free. All the participants signed written informed consents form when the purpose and nature of all the procedures were fully explained prior to the study.

### Subjects

A prospective, cross-sectional study was conducted between January 2016 and December 2019. We examined 85 euthyroid Caucasian women: 39 subjects with HT and 46 control women of similar age. The length of the cycle was from 28 to 35 in control and HT group. HT was diagnosed based on the elevated serum levels of TPOAbs and/or TgAbs combined with the parenchymal heterogeneity found on thyroid ultrasonography. We included subjects with TSH and thyroid hormones within normal ranges during last year. Women with HT were recruited from the Outpatient Department of Endocrinology. Control subjects were recruited among the students and *via* advertisements. All women were non-smoking. The exclusion criteria were: age above 35 years, menstrual irregularity, infertility, polycystic ovary syndrome, pregnancy and breastfeeding; other autoimmune disease (type 1 diabetes, rheumatoid arthritis) and use of hormonal contraception, history of previous surgery, radiotherapy or other medical treatment which could decrease ovarian reserve. Women with HT were not treated with hormonal replacement.

### Study Protocol

The study protocol was the same for HT patients and healthy women. All laboratory studies were performed in the morning, after an overnight fast, during the early follicular phase (3^rd^–5^th^ day) of their menstrual cycles.

### Body Composition Analyses

Body mass index was calculated as the body weight in kilograms divided by the height in meters squared (kg/m^2^).

The percentage of body fat mass were estimated by bioelectric impedance analysis using the InBody 720 (Inbody Co., LTD, Seoul, Korea).

Dual-energy X-ray absorptiometry (DXA) analyses were performed using DXA (GE Healthcare Lunar) at Clinical Research Centre, Medical University of Bialystok by qualified physicians as previously described (27). With this method, body composition with the assessment of fat in the android and gynoid region was estimated. CoreScan software estimated visceral adipose tissue (VAT) mass within the android region.

### Biochemical Analyses

Serum TSH concentration was measured with the immunoradiometric method (sensitivity 0.025 µIU/ml; intra-assay coefficient of variation (CV) – 0.6%; inter-assay CV – 2.1%), and serum free T3 (fT3) (sensitivity 0.3 pg/ml; intra-assay CV- 6.4%; inter-assay CV- 5.5%) and the serum free T4 (fT4) (sensitivity 0.03 ng/dl; intra-assay CV- 10.3%, inter-assay- CV 7.6%) concentrations were detected with radioimmunoassay kits (all DIAsource ImmunoAssays S.A., Belgium). Euthyroidism was defined as having normal levels of TSH (reference range, 0.35-4.5 µIU/ml), fT3 (reference range, 2.3-4.2 pg/ml), and fT4 (reference range, 0.89-1.76 ng/dl). The concentrations of TPOAbs were measured with radioimmunoassay kits (ThermoFisher Scientific, Germany) (sensitivity 5.5 U/mL; intra-assay CV – 3.9%; inter-assay CV – 4.1%) and they were considered positive if their levels exceeded 60 IU/mL. The concentrations of TgAbs were measured with ELISA kits (Euroimmun Medizinische Labordiagnostika G, Germany) (sensitivity 5.5 IU/mL; intra-assay CV – 2.6%; inter-assay CV – 2.5%) and they were considered positive if their levels exceeded 100 IU/mL. Serum FSH and luteinizing hormone (LH) concentrations were measured by immunoradiometric method (DIAsource ImmunoAssays S.A., Belgium). The intra-assay and inter-assay coefficients of variation for LH was below 3.9% and 8%, for FSH below 2% and 4.4%. Serum AMH concentrations were determined by AMH Gen II ELISA enzyme immunoassay kit (Beckmann Coulter, Czech Republic). The lowest concentration of AMH detectable with a 95% probability was 0.08 ng/ml. The intra-assay and inter-assay CV were below 5.4% and 5.6%, respectively. Serum leptin concentrations were measured using an immunoenzymatic method (BioVendor, Czech Republic) (minimum detectable concentration – 0.2 ng/ml; intra-assay CV – 5.9%, inter-assay CV – 5.6%).

### Statistical Analysis

The statistical analyses were performed using Statistica 13.3 package (Statsoft, Cracow, Poland). The variables were tested for normal distribution using Shapiro–Wilk test. Due to non-normal distribution of the data, non-parametric tests were applied, and all values were expressed as median and interquartile range. The comparisons between the women with HT and control group were performed by Mann-Whitney U test. Spearman test was used for correlation analysis. A p-value <0.05 was considered statistically significant.

## Results

**Table 1** presents the main clinical and biochemical characteristics of the studied groups.

**Table 1:** Clinical and biochemical characteristics of the studied groups.

	Control group (n = 46)	Hashimoto thyroiditis (n = 39)	p value
Age (years)	26 (25-27)	26.8 (24-29)	0.76
BMI (kg/m^2^)	21.5 (21-23.8)	21.9 (20.4-25)	0.31
% body fat	26.2 (23-32.9)	32.9 (25.1-35.5)	0.01
Android fat mass (kg)	1.193 (0.797-1.896)	1.148 (0.863-1.821)	0.64
Gynoid fat mass (kg)	4.0 (2.987-4.588)	3.869 (3.192-5.835)	0.5
VAT (g)	168 (89-418)	173 (60-437)	0.83
TSH (uIU/ml)	1.9 (1.4-2.3)	2.3 (1.5-2.9)	0.09
fT4 (ng/dl)	1.2 (1.1-1.3)	1.3 (1.1-1.4)	0.13
fT3 (pg/ml)	3.2 (2.5-3.7)	3.5 (3.1-3.7)	0.52
TPOAbs (IU/ml)	16 (10.5-21.7)	140 (80-744)	<0.01
TgAbs (IU/ml)	8 (5-10)	58.3 (11.9-276.1)	<0.01
LH (IU/l)	4.4 (3.5-5.7)	5.0 (3.5-6.6)	0.11
FSH (IU/l)	5.8 (4.4-7.0)	5.5 (4.1-6.8)	0.57
AMH (ng/ml)	6.3 (4.5-8.6)	4.7 (3-7.5)	0.01
Leptin (ng/ml)	8.4 (4.2-13.9)	10.8 (5.3-16.5)	0.28
TV (ml)	9.5 (7.5-11.5)	9.7 (8.5-13.3)	0.49

Values are expressed as median (interquartile range).

BMI, body mass index; TSH, thyroid−stimulating hormone; fT4, free T4; fT3, free T3; LH, luteinizing hormone; FSH, follicle-stimulating hormone; AMH, anti-Müllerian hormone; VAT, visceral adipose tissue; TPOAbs, thyroid peroxidase antibodies; TgAbs, antibodies against thyroglobulin; TV, thyroid volume.

We did not observe differences in age between the studied groups (p=0.76). No significant differences were found in terms of thyroid function tests (TSH, fT3 and fT4) and thyroid volume in HT women in comparison to the control group (all p>0.05) (**Table 1**).

Women with HT did not differ significantly in BMI when compared to the control group (p=0.31). However, women with HT were characterized by higher % body fat (p=0.01) estimated by bioimpedance method in comparison to the control group. Additionally, in DXA method, we did not find differences in android fat mass, gynoid fat mass and VAT between the studied groups (all p>0.05) (**Table 1**).

We observed lower serum concentration of AMH in HT in comparison to the control group (p=0.01). However, we did not observe difference in serum concentration of leptin between women with HT and the control group (p=0.28) (**Table 1**).

**Figure 1:**
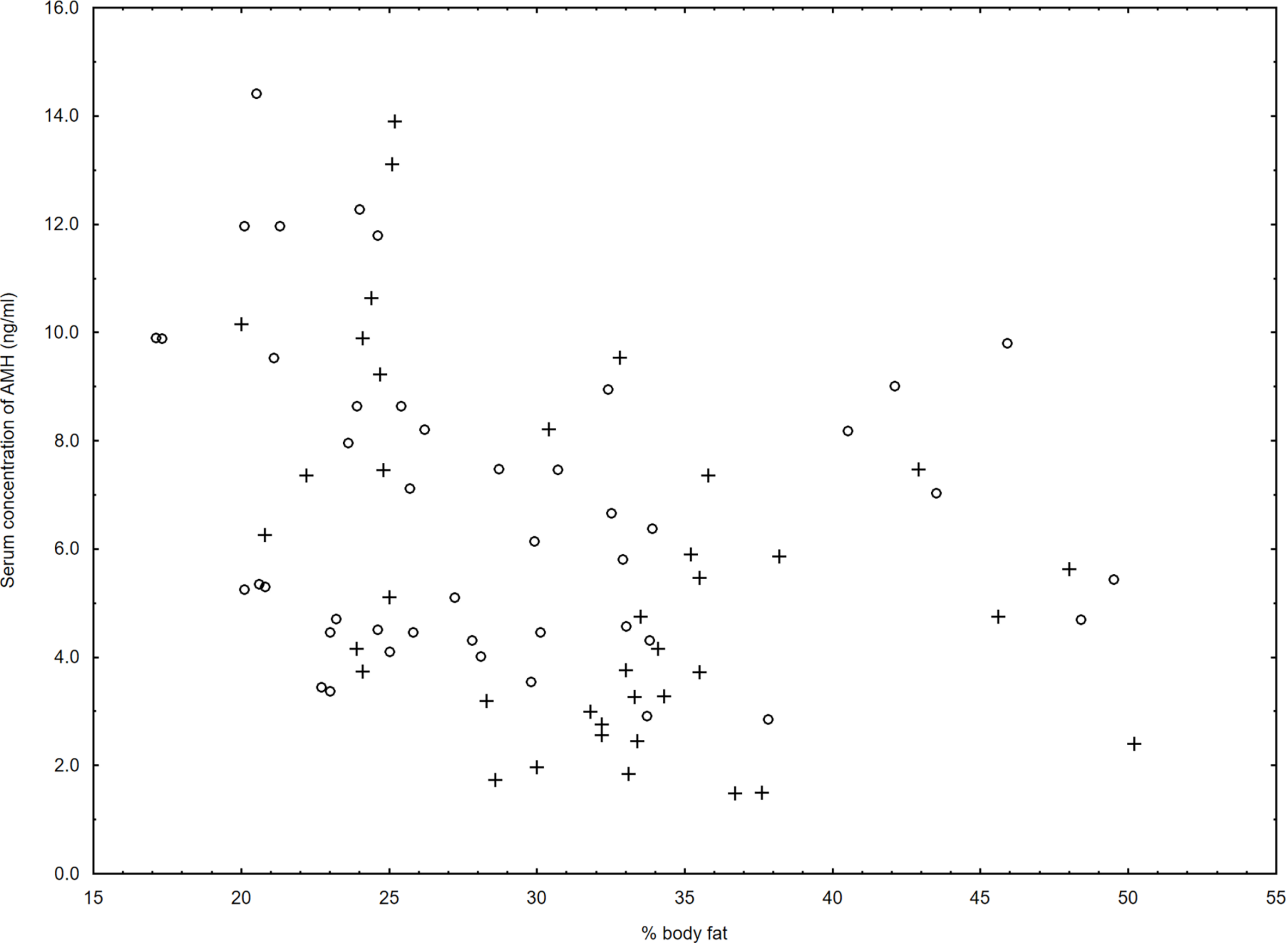
Relationship between serum levels of AMH and % body fat in the women with Hashimoto thyroiditis (r=-0.38, p=0.03) (+) and in the control group (r=-0.19, p=0.14) (○).

We noticed a negative relationship between serum concentration of AMH and % body fat mass (r=-0.38, p=0.03) in women with HT but not in control group (r=-0.19, p=0.14) (**Figure 1**).

Interestingly, we observed a positive relationship between serum levels of AMH and fT3 only in HT group (r=0.33, p=0.04). However, we did not observe relationships between serum concentration of AMH and TSH, fT4, TPOAbs or TgAbs in HT women, nor in the control group (all p>0.05) (**Table 2**).

**Table 2:** The relationship between clinical, biochemical, and hormonal parameters and serum AMH concentration in Hashimoto thyroiditis patients and the control group.

	Control group (n = 46)	Hashimoto thyroiditis (n = 39)
	AMH	
Age (years)	r=-0.19, p=0.18	r=-0.29, p=0.08
BMI (kg/m^2^)	r=0.04, p=0.76	r=0.13, p=0.44
Android fat mass (kg)	r=-0.17, p=0.27	r=0.01, p=0.97
Gynoid fat mass (kg)	r=-0.05, p=0.73	r=0.14, p=0.39
VAT (g)	r=-0.06, p=0.68	r=-0.09, p=0.6
TSH (uIU/ml)	r=-0.08, p=0.56	r=-0.01, p=0.95
fT4 (ng/dl)	r=-0.1, p=0.96	r=-0.1, p=0.54
fT3 (pg/ml)	r=0.07, p=0.6	r=0.33, p=0.04
TPOAbs (IU/ml)	r=0.2, p=0.1	r=0.18, p=0.28
TgAbs (IU/ml)	r=-0.14, p=0.41	r=-0.1, p=0.41
Leptin (ng/ml)	r=-0.15, p=0.41	r=0.01, p=0.96

Data are derived from Spearman correlation coefficient. The level of significance was accepted at p < 0.05.

BMI, body mass index; TSH, thyroid−stimulating hormone; fT4, free T4; fT3, free T3; AMH, anti-Müllerian hormone; TPOAbs, thyroid peroxidase antibodies; TgAbs, antibodies against thyroglobulin.

Additionally, we did not observe relationships between serum levels of AMH and leptin in HT group (r=0.01, p=0.96) (**Table 2**).

We have noticed an association between serum levels of leptin and BMI, % body fat, android fat mass, gynoid fat mass and VAT mass in HT as well as in the control group (all p<0.01) (**Table 3**).

**Table 3:** The relationship between clinical, biochemical, and hormonal parameters and leptin serum concentration in Hashimoto thyroiditis patients and the control group.

	Control group (n = 46)	Hashimoto thyroiditis (n = 39)
	Leptin	
Age (years)	r=-0.17, p=0.35	r=-0.17, p=0.35
BMI (kg/m^2^)	r=0.75, p<0.01	r=0.75, p<0.01
% body fat	r=0.80, p<0.01	r=0.73, p<0.01
Android fat mass (kg)	r=0.77, p=<0.01	r=0.87, p<0.01
Gynoid fat mass (kg)	r=0.82, p=<0.01	r=0.82, p<0.01
VAT (g)	r=0.61, p=<0.01	r=0.64, p<0.01
TSH (uIU/ml)	r=-0.01, p=0.93	r=-0.01, p=0.93
fT4 (ng/dl)	r=-0.6, p=0.74	r=0.24, p=0.19
fT3 (pg/ml)	r=-0.1, p=0.92	r=-0.1, p=0.97
TPOAbs (IU/ml)	r=-0.01, p=0.73	r=-0.01, p=0.92
TgAbs (IU/ml)	r=0.3, p=0.17	r=0.3, p=0.17

Data are derived from Spearman correlation coefficient. The level of significance was accepted at p < 0.05.

BMI, body mass index; VAT, visceral adipose tissue; TSH, thyroid−stimulating hormone; fT4, free T4; fT3, free T3; TPOAbs, thyroid peroxidase antibodies; TgAbs, antibodies against thyroglobulin.

We did not find association between serum concentration of leptin and serum levels of TSH, fT3, fT4, TPOAbs or TgAbs in HT and control group (all p>0.05) (**Table 3**).

## Discussion

In the present preliminary study, we observed lower serum levels of AMH and higher % body fat mass estimated by bioimpedance method in euthyroid women with HT when compared to the control group. Additionally, for the first time, we reported a negative association between serum concentration of AMH and % of body fat mass in women with HT.

In our study, we observed lower serum levels of AMH in euthyroid Caucasian women with HT in comparison to the control group. This is an interesting observation, although the mechanism of this result is not well elucidated. There is a concept that organ specific and non-specific autoantibodies could attack human ovaries (28, 29). It has been found that anti-thyroid antibodies are present in ovarian follicular fluid in euthyroid women with HT, which may exert cytotoxic effect on antral follicles (30). However, in our study, we did not observe an association between serum levels of AMH and TPOAbs or TgAbs. In the literature, conflicting findings regarding the association between serum levels of TPOAbs and AMH are presented: some authors observed a positive relationship (12), whereas others did not find this association (31). Additionally, Polyzos et al. did not observe a connection between the prevalence of positive TPOAbs and high, normal or low functional ovarian reserve (5). Therefore, it has been pointed out that HT was not connected with reduced functional ovarian reserve, but rather with low age-specific AMH levels (5). However, in the last-mentioned study, the authors did not examine serum levels of TgAbs and did not perform USG of thyroid gland, which are in the criteria of diagnosing HT (17). Interestingly, Rao et al. observed that women with subclinical hypothyroidism presented lower indirect ovarian reserve markers, e.g. AFC and serum levels of AMH, and higher FSH serum concentration. Additionally, the authors divided their group according to age and have found that at the age of 35 or more, subclinical hypothyroidism was connected with AFC and serum levels of FSH, AMH, whereas in women at the age of less than 35, subclinical hypothyroidism was only associated with higher serum concentration of FSH (18). It has been established that serum concentration of AMH varies during women’s lifetime, decreasing progressively with age and becoming undetectable at menopause (8). Therefore, the strength of our study is the fact that we included women of comparable age and excluded women older than 35 years, therefore excluding the influence of age on the functional ovarian reserve in our study. In the present study we did not observed differences in serum levels of FSH between women with and without HT. It could be connected with the fact that we examine relatively young women, therefore we did not noticed differences in serum levels of FSH between studied groups. In the previous study higher serum levels of FSH was observed in subclinical HT (18), however we examine euthyroid women with HT *vs* control group. Our finding are consistent with another study that found no significant differences in the basal FSH concentrations between women with TPOAbs negativity and positivity (32). In another study, no differences in serum levels of AMH were observed in the group of adolescent girls with HT in comparison to the control group (31). The explanation of this finding could be connected with the fact that it is too early for the impairment of functional ovarian reserve by autoimmune process in adolescent girls with HT. However, Pirgon et al. observed higher serum levels of AMH in adolescent girls with HT in comparison to the control group (13).

As it was mentioned in the *Introduction*, serum concentration of AMH is considered as one of the most sensitive indicators of functional ovarian reserve, and therefore we can speculate that lower serum levels of AMH in HT women *vs* control group could be connected with decrease of the number of small growing follicle in women with HT, however, this needs to be confirmed by AFC. However, the clinical relevance of these observations remains to be determined. It has been shown that serum levels of AMH and AFC can be useful as predictors of oocyte yield following controlled ovarian stimulation and oocyte retrieval (1, 6). Magri et al. have demonstrated that in women with a good functional ovarian reserve, as assessed by high AMH serum levels, the presence of HT impairs the outcome of controlled ovarian hyperstimulation (COH) (33). Accordingly, in a previous study, it has been observed that women with autoimmune thyroid diseases, as compared to control group, have a poorer ovarian response to gonadotropins (34). Moreover, it has been presented that oocyte fertilization, grade A embryos, and pregnancy rates were lower in women with thyroid autoimmunity *vs* controls, while early miscarriage rate was higher (30). Therefore, based on our study, we can speculate that lower levels of AMH in women with HT can be connected with lower ovarian response to ovarian hyperstimulation in comparison to control group (6). In the face of the fact that the presence of autoimmune thyroid disease impairs the outcome of COH, at least when standard doses of recombinant gonadotropins are used, a pre-stimulation screening for thyroid autoimmunity should be performed even in euthyroid women.

In our study, we observed that euthyroid women with HT were characterized by higher % of body fat estimated by bioimpedance method in comparison to control group, although we did not notice differences in BMI, and body composition estimated by DXA method (android fat mass, gynoid fat mass and VAT mass) between the studied groups. In previous studies, higher percentage of fat has been observed in hypothyroidism as compared to euthyroid controls (35). In another study, Mousa et al. did not observe differences in visceral fat in HT women in comparison to the control group (21). Interestingly, contrary to our findings, they observed that euthyroid women with HT did not differ from the control group in terms of fat percentage (21). However, in this work the authors examined older groups (mean age: control group 39.79 ± 10.42 years; HT group: 41.56 ± 11.68 years) and with higher BMI (control group 28.8 ± 5.35 kg/m^2^; HT group: 27.57 ± 5.20 kg/m^2^) in comparison to our study. We can therefore argue that BMI and age could have impact on the obtained difference between our study and the above-mentioned study.

We observed a negative relationship between serum levels of AMH and % of body fat mass only in women with HT. In the literature, there is no data focused on this aspect, and the studies assessing the relationship between serum levels of AMH and BMI are obscure, especially in the HT group (11–13). Additionally, published studies have shown contradictory results regarding the association between serum levels of AMH and BMI in different group of patients without HT (5, 36–38), where TPOAbs and/or TgAbs were not measured. A positive relationship between serum levels of AMH and BMI in HT group has been reported (13), although this study examined adolescent girls, and the researchers did not assess percentage of fat mass; therefore, it is unclear if this association is connected with fat or fat free mass. In another study, a relationship between serum levels of AMH and BMI in HT was not confirmed, and the authors did not examine body composition in the studied group (12). However, Kucukler et al. found negative relationships between serum level of AMH and BMI in the group of patients consist of subclinical hypothyroidism (n = 21), overt hypothyroidism (n = 21) and controls (n = 32) (11). Interestingly, it has been suggested that an inverse relationship between serum levels of AMH and BMI could be the result of hormone dilution due to higher blood volume in women with elevated BMI (39). As it was mentioned previously, we examined Caucasian women, our groups did not differ in BMI and we included women younger than 35. Therefore, we can exclude the above-mentioned confounding factors. Additionally, in our study, we did not observe an association between serum levels of AMH and BMI in HT women but we found the relationship between serum levels of AMH and % of body fat. However, the possible mechanism of these findings is not clear and needs further comprehensive studies. It has been shown that serum AMH levels were 65% lower in obese women compared to non-obese healthy late reproductive age women (16) and it has been suggested that insulin resistance development related to obesity impairs granulosa cell function and declines circulating AMH levels. However, in our study we did not estimate insulin sensitivity markers. Additionally, it should be take into account that lower serum AMH levels may reflect a lower follicle count due to increased apoptosis or due to lower AMH expression. Based on our results, which showed a connection between the percentage of body fat and serum levels of AMH in women with HT, we can speculate that a decrease in fat mass may have impact on follicle dynamics and growth of the growing follicle pool. That is why behavioural interventions, e.g. exercise and proper diet, could be recommended for euthyroid women with HT. However, prospective data focused on the impact of changes in body composition and serum concentration of AMH in HT women should be performed.

As it was mentioned in the *Introduction*, leptin may play a role in the interaction between thyroid hormones and body composition (24) and can be connected with thyroid autoimmunity (25). However, in our study we did not observe a difference in serum concentration of leptin between women with HT and the control group and we did not find association between serum concentration of leptin and serum levels of thyroid hormones, TPOAbs or TgAbs in HT and control group. In the present study, we observed an association of % body fat, android fat mass and gynoid fat mass with serum levels of leptin in women with HT, as well as in the control group. Therefore, based on obtained results, we cannot confirm the hypothesis about the connection between HT and serum levels of leptin. It should also be noted that we have shown a higher % body fat mass in the HT patients vs control group but it was not reflected by a difference in serum levels of leptin between studied groups. This could be partly explained by the lack of difference between android fat mass, gynoid fat mass and VAT between women with and without HT, which could be the source of leptin. It has been shown that leptin mRNA levels were higher in subcutaneous than in omental adipocytes and leptin mRNA appears to be expressed predominantly by subcutaneous adipocytes (40). ln addition, it has been suggested that smaller adipocytes are characterized by lower expression of the OB gene and secretion of leptin compared to larger fat cells (41). Accordingly, in the previous study conducted by Sieminska and colleagues it has been shown that serum levels of leptin were not altered in women with HT (42). They also indicated that serum leptin levels correlated positively with BMI and waist to hip ratio in HT group and all of the studied women. It should also be emphasized that the secretion of leptin may depend on other factors not related to the adipose tissue depot. It has been observed the large fluctuations in serum leptin concentrations in the presence of relatively small changes in body weight or caloric intake; negative caloric balance could be a signal to the body to reduce leptin production so that appetite would not be inhibited (43). Moreover serum levels of leptin was inversely associated with physical activity (44). Therefore all of this factor should be taken into a count during interpretation of the obtained data. As was mentioned previously our finding suggests that the pathogenesis of lower levels of AMH in HT is connected with fat mass, however is independent of serum concentration of leptin. However, we cannot exclude the impact of the small sample size and complex interactions between leptin, thyroid hormones and body composition on the obtained results.

The main limitation of the present study is a relatively small size of the groups. Additionally, we did not investigate other methods of assessment of functional ovarian reserve, e.g. AFC, inhibin B however, AMH has been proposed to be the most reliable (6).

## Conclusions

On the basis of the obtained results, we concluded that women with HT are characterized by decrease serum levels of AMH and it is associated with higher fat mass, independently of serum levels of leptin. It could mean that women with HT have decreased antral follicle number, however this needs to be confirmed by AFC.

## Data Availability Statement

The raw data supporting the conclusions of this article will be made available by the authors, without undue reservation.

## Ethics Statement

The studies involving human participants were reviewed and approved by Institutional Review Board (Ethics Committee of Medical University of Białystok, Białystok, Poland, approval no. R-I-002/300/2015). The patients/participants provided their written informed consent to participate in this study.

## Author Contributions 

AA: the conception and design of the study, acquisition of data, analysis and interpretation of data. All authors contributed to the analysis and interpretation of data. All authors contributed to the article and approved the submitted version.

## Funding

This work was supported by a Research Grant of Diabetes Poland for a research project submitted as part of the “Competition for a Research Grant of Diabetes Poland”. This study was undertaken with the use of equipment purchased by Medical University of Białystok as a part of the OP DEP 2007-2013, Priority Axis I.3, contrast No POPW.01.03.00-20-008/09.

## Conflict of Interest

The authors declare that the research was conducted in the absence of any commercial or financial relationships that could be construed as a potential conflict of interest.

## Publisher’s Note

All claims expressed in this article are solely those of the authors and do not necessarily represent those of their affiliated organizations, or those of the publisher, the editors and the reviewers. Any product that may be evaluated in this article, or claim that may be made by its manufacturer, is not guaranteed or endorsed by the publisher.
